# Internet Gaming Disorder and Sleep Quality among Jordanian University Students: A Cross-sectional Study

**DOI:** 10.2174/0117450179310269240820042452

**Published:** 2024-08-28

**Authors:** Mahmoud Abdallat, Mohammad Al-Sanouri, Suhayb Al-Salaymeh, Mohammad Zoubi, Tamer Barakat, Ahmad Badwan, Abdallah Alzubi, Rand Murshidi

**Affiliations:** 1 Department of Neurosurgery, The University of Jordan, Amman, Jordan; 2 School of Medicine, The University of Jordan, Amman, Jordan; 3 Department of Dermatology, School of Medicine, The University of Jordan, Amman, Jordan

**Keywords:** Internet gaming disorder, Sleep quality, University students, Gaming disorder, Pittsburgh Sleep Quality Index, Psychiatric conditions

## Abstract

**Background:**

Internet gaming disorder is defined as “Persistent and recurrent use of the internet to engage in games, often with other players, leading to clinically significant impairment or distress.” It is a new evolving disorder that affects many life aspects; therefore, it needs further investigation among different population groups. IGD was introduced for the first time in 2013 in the fifth edition of the *Diagnostic and Statistical Manual of Mental Disorders*, and it suggested carrying out further research among different populations. In 2018, Gaming Disorder (GD) has officially become a type of addiction as the World Health Organization released the 11th revision of the International Classification of Diseases (ICD-11).

**Aims:**

The objective of this study is to investigate the prevalence of internet gaming disorder (IGD) and its association with sleep quality and academic performance among Jordanian university students aged 18-26. Our literature review revealed a lack of research on this topic concerning this specific population and culture. Therefore, our study aims to contribute to the existing literature and to provide insights that can inform prevention, assessment, and treatment strategies for those affected.

**Methods:**

A cross-sectional study design was used by employing convenience and snowball sampling; a total of 2473 participants completed an electronic self-administered questionnaire that included the Internet Gaming Disorder Scale-SF (IGDS9-SF) and Pittsburgh Sleep Quality Index (PSQI). Of these, 432 were excluded based on our criteria. Our inclusion criteria required participants to be Jordanian university students between the ages of 18 and 26, enrolled as undergraduates at Jordanian universities, and free of neurological or psychiatric conditions.

**Results:**

The prevalence of IGD in this study was 15.2% and was more prevalent among males compared to females (p= <0.001). Poor sleep quality was reported by 64.6% of the study population and was more frequently observed in females. Age and academic achievements (GPA) were not associated with having IGD. When binary logistic regression was used, IGD (OR=1.882) positively predicted poor sleep quality.

**Conclusion:**

Internet gaming disorder is common among Jordanian university students and is associated with poor sleep quality, and our findings have significant implications for policymakers, educators, and healthcare providers in raising awareness about the IGD and its impact on sleep quality.

## 1 INTRODUCTION

Video gaming is an emerging form of entertainment that is easily accessible and available to almost everyone nowadays. According to The American Psychiatric Association (APA), around 160 million American adults play internet-based games, and 97% of American children and adolescents play video games for at least 1 hour [1]. This popularity and extensive usage of video games raise concerns about their negative and positive effects on society and individuals’ physical and mental health. Interestingly, specific terms and disorders have been linked to video games, such as Internet Gaming Disorder, excessive video gaming, and even video game addiction, which is similar to other behavioral addictions, such as substance abuse [2].

This research paper revolves around Internet Gaming Disorder (IGD), which is defined as “Persistent and recurrent use of the internet to engage in games, often with other players, leading to clinically significant impairment or distress.” Despite the debate and criticism among researchers on whether to include it or not [3, 4], IGD was first introduced in 2013 in the fifth edition of the *Diagnostic and Statistical Manual of Mental Disorders*, which suggested carrying out further investigation among different populations [5]. In 2018, Gaming Disorder (GD) officially became a type of addiction as the World Health Organization released the 11th revision of the International Classification of Diseases (ICD-11) [6].

Previous researchers have reported significant variations in the prevalence rates of IGD across many countries. According to a meta-analysis of 27 studies from 2007 to 2016, the overall prevalence of IGD ranges between 0.7% and 15.6% [7]. Another meta-analysis of 16 studies on the adolescent population showed a prevalence of 4.6% [8]. However, IGD prevalence is highly variable among different populations, countries, and assessment tools. It reached 38.2% in a sample of 555 Brazilian secondary school students and undergraduates [9]. Another study involving 6000 young adults aged 18-25 years in the United States revealed a prevalence of 24.33% [10]. A different representative study in seven European countries involving 12,938 adolescents aged 14-17 years showed that only 1.6% of the participants have IGD [11]. In Germany, the prevalence of IGD is 1.16% among 11.003 participants aged 13-18 years [12], while in Lebanon, it was 9.2% among 524 participants aged 15–19 years [13].

Video games have been linked to positive social, motivational, cognitive, and emotional effects [1], and improving visual short-term memory [14]. Adolescents with IGD also displayed increased brain activity in the parietal lobe and left orbitofrontal cortex [15]. Despite these benefits, many negative impacts have been associated with IGD including insomnia, depression, anxiety, psychoticism, lower family and extra-family relationships, decreased cognitive control, and increased prefrontal cortex inhibition. Additionally, changes in gray matter volume in different areas of the brain may impair craving and self-control, while alterations in brain networks can contribute to the development and maintenance of IGD and impulse control issues [16-23]. The severity of IGD has also been associated with greater psychological distress and poorer sleep quality [24]. Students with IGD had a higher incidence of suicidal thoughts and self-harm and a higher likelihood of smoking [25-27]. Interestingly, one study showed that adolescents with IGD can affect their sibling’s psychological health and sleep [28].

Many countries have reported a high prevalence of sleep disturbances in our youth, raising international concerns about adolescent sleep health [29]. Poor sleep quality has significant adverse effects on well-being and overall quality of life, including one’s ability to function throughout the day, health problems, perception of pain, the general perception of one’s health and vitality, social functioning, and mental health [30]. Several studies have linked IGD with sleep problems. One study showed that 28.2% of internet gamers had sleep problems in Singapore [31]. A meta-analysis of 33 studies among 26 countries and more than 50,000 participants found that problematic gaming is significantly associated with shorter sleep duration, poorer sleep quality, daytime sleepiness, and sleep problems [32].

The objective of this study is to investigate the prevalence of internet gaming disorder (IGD) and its association with sleep quality and academic performance among Jordanian university students aged 18-26. Our literature review reveals a lack of research on this topic in Jordan concerning this specific population and culture. Therefore, our study aims to contribute to the existing literature and to raise awareness about the impact of IGD on two main aspects of university students' lives: their sleep quality and academic performance.

## 2 METHODS

### 2.1 Study Design

A cross-sectional study was conducted to investigate the relationship between sleep quality and Internet Gaming Disorder among Jordanian university students. An electronic self-administered questionnaire using Google Forms was utilized in the study.

Furthermore, using convenience and snowball sampling, 2437 participants completed the questionnaire. Four hundred thirty-two participants were excluded from our study, as our inclusion criteria required Jordanian university students to be between the ages of 18 and 26, enrolled as undergraduates at Jordanian universities, and free of neurological or psychiatric conditions.

The sample size calculation was conducted *via* an online calculator called Raosoft, with a margin of error of 5% and a confidence level of 95%, and the minimum recommended sample size was 384. Since this study is expected to be generalized to Jordanian university students aged (18-26), we drew our sample from Jordanian universities, with a student population estimated to be around 332,413 [33].

### 2.2 Instrument and Measurement

Our questionnaire included a demographic and background information part and the Arabic versions of the Internet Gaming Disorder Scale-SF (IGDS9-SF) as well as, Pittsburgh Sleep Quality Index (PSQI). The demo- graphics and background information part included questions related to age, gender, education level, academic performance (GPA), time spent on internet gaming per day in the past month, the purpose of their Internet use, and time spent on social media per day in the past month. Moreover, to minimize the effects of other extraneous factors that may affect the sleep quality in this group, participants with a self-reported diagnosis of psychiatric problems or neurological disease were excluded from the study.

#### 2.2.1 The Pittsburgh Sleep Quality Index (PSQI)

The sleep quality was assessed using the Pittsburgh Sleep Quality Index (PSQI). PSQI consists of 19 self-rated questions and five questions rated by the bed partner or roommate. The latter five questions are used for clinical information only, are not tabulated in the scoring of the PSQI, and are not reported on in this article. The 19 self-rated questions assess a wide variety of factors relating to sleep quality, including estimates of sleep duration and latency and of the frequency and severity of specific sleep-related problems. These 19 items are grouped into seven component scores, each weighted equally on a 0-3 scale. The seven component scores are then summed to yield a global PSQI score, which has a range of 0-21, with higher scores indicating worse sleep quality [34]. The PSQI has a high test-retest reliability and good validity for patients with primary insomnia, and it was tested on 80 patients with primary insomnia and 45 healthy control group; a global score > 5 resulted in a sensitivity of 98.7 and specificity of 84.4 as a marker for sleep disturbances in insomnia patients *versus* controls, and the test-retest reliability was 87% [35]. The Arabic version of this questionnaire used in our study was translated by 10 Arabic bilingual translators and tested on 35 healthy Arabic bilinguals, and it revealed moderate internal consistency with a Cronbach’s alpha of 65% and moderate to high correlations between PSQI components and the overall global score [36].

#### 2.2.2 Internet Gaming Disorder Scale- short Form (IGDS9-SF)

The 9-item IGDS9-SF assesses the severity of Internet Gaming Disorder (IGD) and its detrimental effects over a 12-month period [37]. After obtaining permission, the Arabic-translated version of the dichotomous scale was used, and it was tested and validated on 204 Egyptian students and produced an appropriate internal consistency (Cronbach alpha = 0.612). The questionnaire consists of 9 yes-or-no questions; the scale assigns one point for each “yes” answer and zero point for each “No,” where a score of (0-2) indicates normal gamers, a score of (3 to 5) indicates a risky gamer, and (6 or more) indicates a disordered gamer [38].

### 2.3 Data Collection\procedure

The link for the questionnaire was shared on Jordanian universities' social media groups, and the participants were asked to share it with other possible eligible participants. The data collection took place in June 2022 for 6 weeks. Participants responded to an online questionnaire that consisted of three parts, with an average duration of 10 minutes to answer all the questions. Participants can enter the survey only after they have provided an online consent form that expresses their willingness to participate. Their responses were confidential. All the questions included in the questionnaire are required to be filled while setting the form to have no missing value. Numerical entry questions did not allow for nonsensical inputs. The collected data was converted into a Microsoft Excel spreadsheet for subsequent analysis by the research team.

### 2.4 Ethical Considerations

The ethical approval was accomplished by filling out the research approval application provided by the institutional review board (IRB) at the University of Jordan (92/2023). In addition, to ensure confidentiality, the questionnaire did not contain any personally identifiable information, such as names, addresses, government-issued identification numbers, or other data that can be easily linked to individuals. Furthermore, all computerized data files were locked in password-protected devices.

### 2.5 Data analysis

All data were entered in Microsoft Excel 2016 and imported to IBM Statistical Package for the Social Sciences 26 (SPSS) for statistical analysis. Descriptive statistics were used to describe the frequencies of sleep quality, IGD, and other socio-economic factors. Continuous data were expressed as mean± standard deviation (SD). Chi-square was used to examine the bivariate correlations between gamer groups and sleep quality and associated factors. The associations between the severities of IGD and sleep quality were further analyzed using logistic regression, which was controlled for potential confounders. Specifically, sleep quality was entered as a dependent variable; IGD status, social media hours per day, and family income were entered as independent variables. Gender was not added for logistic regression analysis due to multicollinearity. A value of p < 0.05 was considered significant for all analysis tests.

## 3 RESULTS

A total of 2437 participants completed the questionnaire. After excluding participants under the age of 18 or above 26, incomplete questionnaires, and participants previously diagnosed with psychiatric or neurological disorders, 2005 participants (82.2%) were included in the study. The study sample had a higher ratio of females (52.5%, n=1053) to males. Most participants (62.7%, n=1258) were between 18-20 years old, and 68.5% (n=1373) were from the middle region of Jordan. The participants attended 30 different universities across Jordan, with the most common being The University of Jordan (28.9%, n=580) and Jordan University of Science and Technology (13.4%, n=269). About one-third of the study population were students of scientific majors (35.6%, n=713), followed by students of health majors and students of humanities majors (35.5%, n=711 and 29%, n=581, respectively). The main reasons for internet use were social media (55.9%, n=1120) and studying (28.3%, n=568). The average gaming and social media hours per day were 1.8 and 5.2 hours, respectively. Detailed demographic information is summarized in Table **1**.

The participants' average IGD score was 2.48 out of 9, with 15.2% (n=305) of the participants having IGD and 25.2% (n=506) of them being risky players. Among The University of Jordan students, the prevalence of IGD and risky gamers were 15% (n=87) and 24.5% (n=142), respectively. IGD and being a risky gamer were more prevalent among males compared to females (p= <0.001), and they were more prevalent among students of scientific majors and humanities majors compared to health majors (p=0.018). Age, region, GPA, family income, living in the countryside, and hours spent on social media were not significantly associated with either IGD status. Detailed information regarding IGD status and its association with demographic factors is shown in Table **2** and Fig. (**1**).

The average PSQI score for our sample is 6.91 out of 24, with 35.4% (n=710) of participants reporting good sleep quality and 64.6% (n=1295) of them reporting poor sleep quality. Among The University of Jordan students, 61.4% (n=356) had poor sleep quality, and 38.6% (n=224) had good sleep quality. A closer examination of sleep components revealed a significant correlation between IGD status and subjective sleep quality, sleep disturbance, use of sleep medication, and daytime dysfunction (p<0.05). More details are shown in Table **3**. Poor sleep quality was significantly more common in females, lower family income groups, participants with IGD, and risky gamers (p<0.05). Fig. (**2**) illustrates PSQI scores distributed by gender and IGD status. Hours spent on social media per day were also significantly associated with sleep quality (p<0.05). Binary logistic regression analysis indicated that being a risky gamer and having IGD (OR=1.397, 1.882 respectively), spending more hours on social media (OR=1.38), and having a lower family income (OR=1.592) were significant predictors of poor sleep quality when adjusted for other variables. Further details are shown in Table **4**.

## 4 DISCUSSION

To the best of our knowledge, this is the first study to investigate IGD prevalence and its association with gender, academic achievements, and sleep quality among Jordanian university students. We found that 15.2% of Jordanian university students have IGD, a high prevalence compared to 9.2% in Lebanon [13], 6.1% among 423 gamers in 3 Arab countries including Jordan [17], 7.1% in Pakistan [39], 5.3% in the US [25], 1.6% among 7 European countries [11], and 4.6% in a Meta-analysis across 3 decades on adolescents aged 10-19 years [8]. A recent meta-analysis conducted on 19 studies in Asia and 3 in Europe found that the pooled prevalence among all ages was 6.7% [40]. Restricting the analysis to students at the University of Jordan, the largest university in the country, showed a similar pattern (15% with IGD and 24.5% being risky gamers). A recent study in a single university in Egypt which shares a similar socio-cultural environment with Jordan found that 6% of the students aged 18-25 years old had IGD [41], this high prevalence in our study may be due to the recruitment of a young age group (18-26), who are more likely to play video games than the general population, as prior studies found that the prevalence of video game addiction decreased with age and being younger is associated with IGD [42, 43]. Additionally, our study was conducted after the COVID-19 pandemic and quarantine measures, unlike many of the studies mentioned, which were conducted before the pandemic. A large study in Japan found that IGD prevalence has increased during the COVID-19 pandemic by 1.6 times compared to before the pandemic [44]. On the other hand, Brazil had a higher IGD prevalence of 38.2% [9] and 19.9% in England [45]. This variability in prevalence can be attributed to different assessment tools and thresholds, as well as different age groups and populations. Although two studies in Pakistan and Slovenia used the same tool we used in our study (IGDS9-SF) in a nearly similar age group (16-28 and 12-16 years old respectively), their IGD prevalence was 1.5% and 2.5%, respectively [46, 47].

In our study, the prevalence of being a risky gamer (25.2%) is higher than the IGD prevalence (15.2%). This is consistent with a study in Pakistan, which showed that 25.2% of the sample were risky gamers and 7.1% had IGD [39]. Similarly, a study in the US showed the same pattern, with 9.2% being risky gamers and 5.3% having IGD [25]. Other studies in Egypt and Lebanon also showed higher percentages of risky gamers compared to those with IGD (50% *vs*. 13.6% in Egypt and 35.7% *vs*. 9.2% in Lebanon) [13, 48]. These results can be explained by needing a lower threshold and lower score to be a risky gamer before having IGD.

Despite the higher number of females who participated in our study (52.5%), we found that IGD is significantly associated with the male gender, with19% of males having IGD compared to 11.8% of females. In contrast, a study at the University of Mansoura in Egypt did not find any significant gender difference in IGD prevalence [41]. Nevertheless, our result aligns with many studies showing higher prevalence in males compared to females. For example, the prevalence of IGD in European adolescent males was 3.1%, and in females was 0.3% [11]. Similar results were demonstrated in a Nationwide German study with a large sample of 44,610 participants [49]. A Canadian study showed that Males were also significantly more likely to have IGD than females (15.1% *vs*. 3.1%, respectively) [50]. Additionally, studies in Slovenia, Germany and Brazil demonstrated that being male is a risk factor for IGD [9, 51, 52]. Another study in Norway showed that males report more problems with gaming than females [43]. Siste *et al*. [53] from Indonesia found that males were 4 times more likely to have internet gaming disorder than females. This result can be explained by the male preference for certain game genres, which themselves are more time-consuming and demanding [52]. Moreover, functional magnetic resonance imaging studies showcased a higher activity in the mesocorticolimbic pathway in males compared to females when playing video games [54]. Males were also more sensitive to gaming-related rewards and higher cravings than females [55]. In addition, males spent more time playing video games. A national study conducted in the US found that the average playtime for males was 16.4 hours per week, compared to 9.2 hours per week for females [56]. Furthermore, our findings showed that increased gaming hours were associated with IGD. These findings parallel the results of previous studies looking into the link between gaming time and IGD risk [57]. Additionally, socio-cultural factors, such as outdoor gaming centers being mostly frequented by males in our country, may contribute to the higher IGD prevalence among males.

GPA was not associated with IGD in our study, in contrast to 2 large studies in Germany [10, 47] and one across 7 European countries [11], which found that students with IGD had lower grades in school and diminished academic performance. This discrepancy can be explained by the fact that 24.3% of the sample were in their first semester at university, so they did not have GPAs yet, which might have affected our results. Age was also not associated with having IGD, similar to a study among European adolescents aged between (14-17) years [11]. This could be due to the narrow age group range (18-26) used in our study, resulting in participants with a similar lifestyle. Moreover, 2 studies in Norway assessed IGD in wide age groups ((15-40) years and (16-74) years respectively) and found that young age is significantly associated with IGD [42, 43].

64.6% of Jordanian university students reported poor sleep quality, which is similar to 60% of Al-Azhar university students [48] and 65.4% of Ethiopian adults [58]. This high prevalence should raise a national concern as sleep disorders play a central role in the development of Dysregulation of mood, energy, and social rhythms syndrome (DYMERS); a newly emerged concept that carries a substantial influence on health-related quality of life and can be a consequence for many disorders such as panic disorder [59, 60].

In comparison to normal gamers, our results showed that participants with IGD and risky gamers had lower subjective sleep quality, sleep disturbance, daytime dysfunction, and use of sleep medication. Previous studies have established a link between pathological gaming and poor sleep quality. IGD has many effects on different components of sleep, and it was linked to sleeping duration [13], sleep disorders and insomnia [61], and daytime tiredness [62]. Furthermore, IGD was associated with poorer sleep quality, consistent with research conducted in France [63], Pakistan [39], and the US [25]. These results can be explained by the fact that students with IGD were more likely to delay their bedtimes and shorten their total time spent sleeping or even wake up during the night to continue gaming [13, 64]. Playing online games triggers the release of norepinephrine in the cortex and dopamine in the midbrain. These neurotransmitters are part of the reticular activating system (RAS) and are responsible for keeping gamers awake [65]. Additionally, light exposure during gaming can suppress melatonin production, a hormone produced by the pineal gland at night and in darkness [66]. Furthermore, for many gamers, staying up late to play can be a socially rewarding experience [67]. All these negative effects of IGD on sleep quality highlight the need for treatment. Two recent systematic reviews found that non-pharmacotherapy modalities such as behavioral therapy (CBT)/multi-level counseling (MLC) and pharmacotherapy treatments such as bupropion, methylphenidate, and a range of selective serotonin reuptake inhibitors are all effective in treating IGD [68, 69].

There are a few limitations associated with this study that should be addressed. This study, as with any cross-sectional study, has an inherent bias as it measures both the exposure and the outcome simultaneously, making it difficult to determine causality. Accordingly, this study cannot support causal links due to the lack of a time dimension. The survey was conducted *via* an online questionnaire, and convenience and snowball sampling were used to collect data, both of which could have resulted in sampling bias. Since questionnaires were distributed to acquaintances who may share common characteristics and participants were chosen based on availability, there is a risk of self-selection bias. Additionally, participants may not have accurate time perception. Our data collection tool is slightly bulky, including more than 40 questions, and some participants may answer some questions carelessly. Further research should explore the long-term effects of IGD on academic performance and mental health among university students. Longitudinal studies could provide deeper insights into the causative factors and effectiveness of various intervention strategies in different ethnic and age groups (Supplementary material-IDG).

## CONCLUSION

Internet Gaming Disorder (IGD) is common among Jordanian university students and is significantly associated with poor sleep quality. Our findings emphasize the need for urgent intervention and targeted strategies to mitigate the effects of IGD on this vulnerable population. Policymakers, educators, and healthcare providers must raise awareness about IGD and its impact on sleep quality. Efforts are necessary to identify individuals at risk and to implement early interventions to protect future generations from this emerging global issue and its consequences, which impose a high burden on society.

## AUTHOR CONTRIBUTION

It is hereby acknowledged that all authors have accepted responsibility for the manuscript's content and consented to its submission. They have meticulously reviewed all results and unanimously approved the final version of the manuscript.

## Figures and Tables

**Fig. (1):**
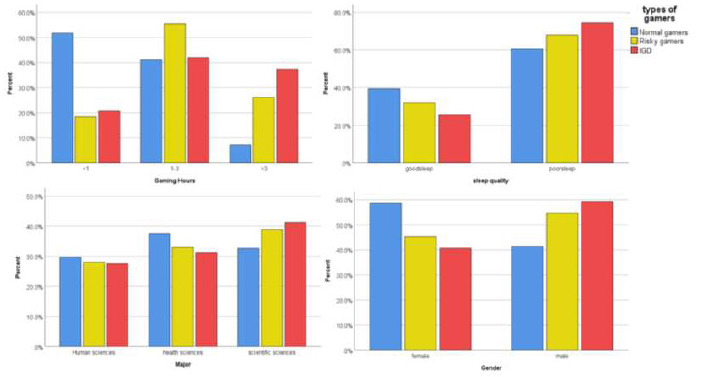
These four bar charts demonstrate the significant associations between gaming group clustering and various demographic and gaming-related variables, with percentages represented on the y-axis.

**Fig. (2):**
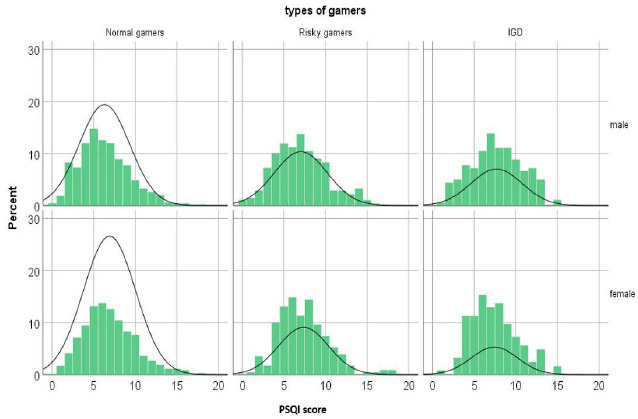
This histogram represents the distribution of PSQI (Pittsburgh Sleep Quality Index) scores, categorized by gender and gaming group, with percentages on the y-axis. The normal distribution curve shows the approximate frequency of each group.

**Table 1:** Demographic data & internet use.

**Characteristics**		-			-	
-		N=2005			(%)	
**Age**		-			-	
18-20		1258			62.7	
21-23		579			28.9	
>23		168			8.4	
**Sex**		-			-	
Female		1053			52.5	
male		952			47.5	
**University**		-			-	
University of Jordan		580			28.9	
Jordan University of Science and Technology		269			13.4	
Hashemite University		275			13.7	
Yarmouk University		117			5.8	
Balqa Applied University		189			9.4	
Mutah University		106			5.3	
Al-Bayt University		105			5.2	
Others		364			18.5	
**Family income**		-			-	
<500		689			34.4	
500-1000		837			41.7	
1000-1500		255			12.7	
>1500		224			11.2	
**Resident**		-			-	
Countryside		449			22.4	
City		1556			77.6	
**Region**		-			-	
North		513			25.6	
Middle		1373			68.5	
south		119			5.9	
**Major**		-			-	
Human sciences		581			29	
Health majors		711			35.5	
Scientific majors		713			35.6	
**Main Internet Use**		-			-	
Social media		1120			55.9	
Studying		568			28.3	
Video Games		103			5.1	
Multiple reasons		202			10.1	
others		12			0.6	
**Social media (hours/day)**		-			-	
0-2		341			17	
3-4		609			30.4	
>4		1055			52.6	
**Gaming hours(hours/day)**		-			-	
<1		773			38.6	
1-3		901			44.9	
>3		331			16.5	

**Table 2:** IGD status and its association with demographic factors.

Characteristics	IGD Status		-	-		-		Chi-square	-
-	Normal Gamer		Risky Gamer	IGD		Total		p-values	χ2
**Age**	n(%)		n(%)	n(%)		-		0.714	2.12
18-20	742(59)		329(26.2)	187(14.9)		1258(62.7)		-	-
21-23	350(29.3)		140(27.7)	89(29.2)		579(28.9)		-	-
>23	102(60.7)		37(22)	29(17.3)		168(8.4)		-	-
**Sex**	-		-	-		-		<0.001	45.78
Female	700(66.5)		229(21.7)	124(11.8)		1053(52.5)		-	-
male	494(51.9)		277(29.1)	181(19)		952(47.5)		-	-
**Family income**	-		-	-		-		0.295	7.29
<500	410(59.5)		170(24.7)	109(15.8)		689(34.4)		-	-
500-1000	514(61.4)		196(23.4)	127(15.2)		837(41.7)		-	-
1000-1500	149(58.4)		69(27.1)	37(14.5)		255(12.7)		-	-
>1500	121(54)		71(31.7)	32(14.3)		224(11.2)		-	-
**Grade**	-		-	-		-		0.182	13.82
First semester	299(61.4)		119(24.4)	69(14.2)		487(24.3)		-	-
Weak	3(37.5)		4(50)	1(12.5)		8(0.4)		-	-
Acceptable	19(47.5)		16(40)	5(12.5)		40(2)		-	-
Good	142(55.5)		73(28.5)	41(16)		256(12.8)		-	-
Very good	428(58.2)		185(25.2)	122(16.6)		735(36.7)		-	-
excellent	303(63.3)		109(22.8)	67(14)		479(23.9)		-	-
**Resident**	-		-	-		-		0.176	3.48
Countryside	254(56.6)		115(25.6)	80(17.8)		449(22.4)		-	-
City	940(60.4)		391(25.1)	225(14.5)		1556(77.6)		-	-
**Region**	-		-	-		-		0.214	5.81
North	307(59.8)		118(23)	88(17.2)		513(25.6)		-	-
Middle	813(59.2)		364(26.5)	196(14.3)		1373(68.5)		-	-
south	74(62.2)		24(20.2)	21(17.6)		119(5.9)		-	-
**Major**	-		-	-		-		0.018	11.88
Human sciences	355(61.1)		142(24.4)	84(14.5)		581(29)		-	-
Health majors	449(63.2)		167(23.5)	95(13.4)		711(35.5)		-	-
Scientific majors	390(54.7)		197(27.6)	126(17.7)		713(35.6)		-	-
**Social media**	-		-	-		-		.636	2.55
0-2	210(61.6)		83(24.3)	48(14.1)		341(17)		-	-
3-4	373(61.2)		147(24.1)	89(14.6)		609(30.4)		-	-
>4	611(57.9)		276(26.2)	168(15.9)		1055(52.6)		-	-
**Gaming hours**	-		-	-		-		<0.001	321.37
<1	617(79.8)		93(12)	63(8.2)		773(38.6)		-	-
1-3	492(54.6)		281(31.2)	128(14.2)		901(44.9)		-	-
>3	85(25.7)		132(39.9)	114(34.4)		331(16.5)		-	-

**Table 3:** Analysis of PSQI components and its association with IGD status.

Sleep Parameters	IGD Status, N(%)	-	-	-	*χ* ^2^	*p*-values
-	Normal Gamers	Risky Gamers	IGD	Total	-	-
**Subjective sleep quality**	-	-	-	-	36.819	<0.001
Very good	249(69.6)	78(21.8)	31(8.7)	358(17.9)	-	-
Fairly good	602(60)	252(25.1)	150(14.9)	1004(50.1)	-	-
Fairly bad	277(56.1)	127(25.7)	90(18.2)	494(24.6)	-	-
Very bad	66(44.3)	49(32.9)	34(2.8)	149(7.4)	-	-
**Sleep latency**(minutes)	-	-	-	-	10.334	0.111
0(better)	301(64.5)	107(22.9)	59(12.6)	467(23.3)	-	-
1	406(58.8)	183(26.5)	101(14.6)	690(34.4)	-	-
2	290(56.3)	140(27.2)	85(16.5)	515(25.7)	-	-
3(worse)	197(59.2)	76(22.8)	60(18)	333(16.6)	-	-
**Sleep duration**	-	-	-	-	10.809	0.094
>7 h	556(58.9)	237(25.1)	151(16)	944(47.1)	-	-
6–7 h	440(57.8)	200(26.3)	121(15.9)	761(38)	-	-
5–6 h	127(63.2)	47(23.4)	27(13.4)	201(10)	-	-
<5 h	71(71.7)	22(22.2)	6(6.1)	99(4.9)	-	-
**Sleep efficiency**	-	-	-	-	4.219	0.647
>85%	734(59.7)	314(25.5)	181(14.7)	1229(61.3)	-	-
75–84%	2.7(62.2)	76(22.8)	50(15)	333(16.6)	-	-
65–74%	140(59.8)	58(27.8)	36(15.4)	234(11.7)	-	-
<65%	113(54.1)	58(27.8)	38(18.2)	209(10.4)	-	-
**Sleep disturbance**	-	-	-	-	29.268	<0.001
0(better)	64(66)	19(19.6)	14(14.4)	97(4.8)	-	-
1	693(63.5)	267(24.5)	132(12.1)	1092(54.5)	-	-
2	390(53.6)	199(27.4)	138(19)	727(36.3)	-	-
3(worse)	47(52.8)	21(23.6)	21(23.6)	89(4.4)	-	-
**Use of sleep medication**	-	-	-	-	9.478	0.009
Not during the past month	1068(60.8)	430(24.5)	258(14.7)	1756(87.6)	-	-
Less than once a month	126(50.6)	76(30.5)	47(18.9)	249(12.4)	-	-
**Daytime dysfunction**	-	-	-	-	49.918	<0.001
0(better)	148(70.1)	44(20.9)	19(9)	211(10.5)	-	-
1	644(63.8)	228(22.6)	137(13.6)	1009(50.3)	-	-
2	326(53.6)	177(29.1)	105(17.3)	608(30.3)	-	-
3(worse)	76(42.9)	57(32.2)	44(24.9)	177(8.8)	-	-

**Table 4:** Association of study variables with sleep quality.

Characteristics	Sleep Quality			Chi-square	-	-	-		-	
-	Good Sleep Quality	Poor Sleep Quality	Total	p-values	χ2	-	-		-	
**Age**	F(%)	F(%)	F(%)	0.448	1.61	-	-		-	
18-20	441(35.1)	817(64.9)	1258(62.7)	-	-	-	-		-	
21-23	215(37.1)	364(62.9)	579(28.9)	-	-	-	-		-	
>23	54(32.1)	114(67.9)	168(8.4)	-	-	-	-		-	
**Sex**	-	-	-	0.026	4.99	-	-		-	
Female	349(33.1)	704(66.9)	1053(52.5)	-	-	-	-		-	
male	361(37.9)	591(62.1)	952(47.5)	-	-	-	-		-	
**Grade**	-	-	-	0.28	6.28	-	-		-	
First semester	161(33.1)	326(66.9)	487(24.3)	-	-	-	-		-	
Weak	0(0)	8(100)	8(0.4)	-	-	-	-		-	
Acceptable	14(35)	26(65)	40(2)	-	-	-	-		-	
Good	93(36.3)	163(63.7)	256(12.8)	-	-	-	-		-	
Very good	270(36.7)	465(63.3)	735(36.7)	-	-	-	-		-	
excellent	172(35.9)	307(64.1)	479(23.9)	-	-	-	-		-	
**Resident**	-	-	-	0.314	1.02	-	-		-	
Countryside	150(33.4)	299(66.6)	449(22.4)	-	-	-	-		-	
City	560(36)	996(64)	1556(77.6)	-	-	-	-		-	
**Region**	-	-	-	0.189	3.33	-	-		-	
North	166(32.4)	347(67.6)	513(25.6)	-	-	-	-		-	
Middle	497(36.2)	876(63.8)	1373(68.5)	-	-	-	-		-	
south	47(39.5)	72(60.5)	119(5.9)	-	-	-	-		-	
**Major**	-	-	-	0.106	4.48	-	-		-	
Human sciences	187(32.2)	394(67.8)	581(29)	-	-	-	-		-	
Health majors	269(37.8)	442(62.2)	711(35.5)	-	-	-	-		-	
Scientific majors	254(35.6)	459(64.4)	713(35.6)	-	-	-	-		-	
Gaming hours	-	-	-	0.854	0.315	-	-		-	
<1	270(34.9)	503(65.1)	773(38.6)	-	-	-	-		-	
1-3	325(36.1)	576(63.9)	901(44.9)	-	-	-	-		-	
>3	115(34.7)	216(65.3)	331(16.5)	-	-	-	-		-	
-	-	-	-	-	-	**Logistic Regression**				
-	-	-	-	-	-	*P*		*OR*		*CI 95%(UL-LL)*
**Family income**	-	-	-	0.016	10.31	0.023	-		-	
<500	227(32.9)	462(67.1)	689(34.4)	-	-	0.003	1.592		1.166-2.174	
500-1000	288(34.4)	549(65.6)	837(41.7)	-	-	0.008	1.507		1.113-2.042	
1000-1500	96(37.6)	159(62.4)	255(12.7)	-	-	0.152	1.309		0.905-1.894	
>1500^(ref)^	99(44.2)	125(55.8)	224(11.2)	-	-	-	-		-	
**Social media(h)**	-	-	-	0.004	10.93	0.014	-		-	
0-2^(ref)^	138(40.5)	203(59.5)	341(17)	-	-	-	-		-	
3-4	233(38.3)	376(61.7)	609(30.4)	-	-	0.515	1.095		0.833-1.441	
>4	339(32.1)	716(67.9)	1055(52.6)	-	-	0.012	1.386		1.075-1.788	
**IGD status**	-	-	-	<0.001	23.61	<0.001	-		-	
Normal gamers^(ref)^	470(39.4)	724(60.6)	1194(59.6)	-	-	-	-		-	
Risky gamers	162(32)	344(68)	506(25.2)	-	-	0.003	1.397		1.119-1.744	
IGD	78(25.6)	227(74.4)	305(15.2)	-	-	<0.001	1.882		1.417-2.499	

## Data Availability

The data supporting the findings of the article is available in the Zenodo Repository at https://zenodo.org/records/13382368, reference number [10.5281/zenodo.13382368].

## LIST OF ABBREVIATIONS

GD: = Gaming Disorder
ICD-11: = International Classification of Diseases
IGD: = Internet Gaming Disorder
PSQI: = Pittsburgh Sleep Quality Index
APA: = American Psychiatric Association
RAS: = Reticular Activating System
MLC: = Multi-Level Counseling

## STANDARDS OF REPORTING

STROBE guidelines were followed.
