# Supplementary material for: Internet Gaming Disorder and Sleep Quality among Jordanian University Students: A Cross-sectional Study
Source: Clin Pract Epidemiol Ment Health. 2024 Aug 28;20:e17450179310269. doi: 10.2174/0117450179310269240820042452 (PMC11748057; doi:10.2174/0117450179310269240820042452)
Supplement: Supplementary file 1 — Supplementary material is available on the publisher's website along with the published article. [file CPEMH-20-E17450179310269_SD1.pdf]

Clin. Pract. Epidemiol. Ment. Health
DOI: 10.2174/0117450179310269240820042452, 2024, 20, e17450179310269

ISSN: 1745-0179

SUPPLEMENTARY MATERIAL

OPEN ACCESS

Internet Gaming Disorder and Sleep Quality among Jordanian University Students: A Cross-sectional Study

Mahmoud Abdallat[1,#], Mohammad Al-Sanouri[2,*,#], Suhayb Al-Salaymeh[2], Mohammad Zoubi[2], Tamer Barakat[2], Ahmad Badwan[2], Abdallah Alzubi[2] and Rand Murshidi[3]

[1]*Department of Neurosurgery, The University of Jordan, Amman, Jordan*
[2]*School of Medicine, The University of Jordan, Amman, Jordan*
[3]*Department of Dermatology, School of Medicine, The University of Jordan, Amman, Jordan*

© 2024 The Author(s). Published by Bentham Open.
This is an open access article distributed under the terms of the Creative Commons Attribution 4.0 International Public License (CC-BY 4.0), a copy of which is available at: https://creativecommons.org/licenses/by/4.0/legalcode. This license permits unrestricted use, distribution, and reproduction in any medium, provided the original author and source are credited.

*Address correspondence to this author at the School of Medicine, The University of Jordan, Amman, Jordan;
E-mail: Mohammad.tarek99@gmail.com
#These authors contributed equally to this work

Published: August 28, 2024

Cite as: Abdallat M, Al-Sanouri M, Al-Salaymeh S, Zoubi M, Barakat T, Badwan A, Alzubi A, Murshidi R. Internet Gaming Disorder and Sleep Quality among Jordanian University Students: A Cross-sectional Study. Clin Pract Epidemiol Ment Health, 2024; 20: e17450179310269. http://dx.doi.org/10.2174/0117450179310269240820042452

Send Orders for Reprints to
reprints@benthamscience.net

Internet Gaming Disorder and Sleep Quality among Jordanian Youth

This questionnaire is an invitation to participate in a study conducted by a research team from the Jordan University Hospital.

This questionnaire consists of three sections and is expected to take less than five minutes to complete.

We encourage you to answer freely without hesitation, as the questionnaire does not require any personal or identifying information.

All results will be treated with complete confidentiality and will not be shared outside the research team.

Participation in this study is voluntary, and you may withdraw from completing the questionnaire at any stage.

There are no benefits or risks associated with filling out this questionnaire.

Your accurate responses will greatly contribute to the success of this research and the accuracy of its results.

Thank you for your valuable time and participation in this research.

For more information or inquiries, please contact Tamer.nmbres@gmail.com.

* Indicates required question

1. If you agree to participate in this study, please click *

Mark only one oval.

- I agree to participate in this study

2. Anyone diagnosed with mental or neurological disorders is excluded from this study. We wish everyone health and well-being. *

Mark only one oval.

- I'm not diagnosed with mental or neurological disorder

Demographics

3. Gender *

Mark only one oval.

- Male
- Female

4. Age *

Mark only one oval.

- 10
- 11
- 12
- 13
- 14
- 15
- 16
- 17
- 18
- 19
- 20
- 21
- 22
- 23
- 24
- 25
- 26
- 27
- 28
- 29
- 30
- 31
- 32
- 33
- 34
- 35
- 36
- 37
- 38
- 39
- 40

5. City *

Mark only one oval.

- Amman
- Zarqa
- Irbid
- Balqa
- Jerash
- Tafila
- Ajloun
- Aqaba
- Karak
- Madaba
- Ma'an
- Mafraq

6. Area of residence *

Mark only one oval.

- Rural
- Urban (City)

7. University *

Mark only one oval.

- University of Jordan
- Jordan University of Science and Technology
- Yarmouk University
- Hashemite University
- Applied Science University
- Al al-Bayt University
- Al-Hussein Bin Talal University
- Balqa Applied University
- Mutah University
- Tafila Technical University
- German-Jordanian University
- Middle East University
- Al-Ahliyya Amman University
- Aqaba University of Technology
- Ajloun National University
- American University of Madaba
- Jadara University
- Amman Arab University
- Zarqa Private University
- Irbid National University
- Al-Zaytoonah University of Jordan
- Jerash Private University
- Princess Sumaya University for Technology
- Al-Isra University
- Petra University
- Applied Science Private University
- Philadelphia University
- Arab Open University
- Al Hussein Technical University
- The World Islamic Science & Education University
- Others

8. Field of study *

Mark only one oval.

- Health Faculties (Medicine, Dentistry, Pharmacy, Doctor of Pharmacy, Nursing, Laboratory Sciences, Rehabilitation Sciences)
- Scientific Faculties (Sciences, Agriculture, Engineering, Information Technology)
- Humanities Faculties (Arts, Business, Sharia, Educational Sciences, Law, Sports Education, Arts and Design, Foreign Languages, International Studies)

9. Grade Point Average (GPA)

____

10. University grade *

Mark only one oval.

- Excellent
- Very Good
- Good
- Acceptable
- Poor
- Still in the first semester

11. Family's monthly income in Dinars

Mark only one oval.

- Less than 500
- 500-1000
- 1000-1500
- More than 1500

12. Main reason for using the internet *

Mark only one oval.

- Social media
- Studies
- Online Games
- Other: ________________

13. During the past month, how many hours did you spend daily on online games? *

Answer in hours per day

Mark only one oval.

- 0
- 1
- 2
- 3
- 4
- 5
- 6
- 7
- 8
- 9
- 10
- 11
- 12
- 13
- 14
- 15
- 16
- 17
- 18
- 19
- 20
- 21
- 22
- 23
- 24

14. During the past month, how many hours did you spend daily on social media? *

Answer in hours per day

Mark only one oval.

- 0
- 1
- 2
- 3
- 4
- 5
- 6
- 7
- 8
- 9
- 10
- 11
- 12
- 13
- 14
- 15
- 16
- 17
- 18
- 19
- 20
- 21
- 22
- 23
- 24

PITTSBURGH SLEEP QUALITY INDEX

The following questions relate to your usual sleep habits during the past month only. Your answers
should indicate the most accurate reply for the majority of days and nights in the past month.
Please answer all questions

15. 1) During the past month, what time have you usually gone to bed at night? *

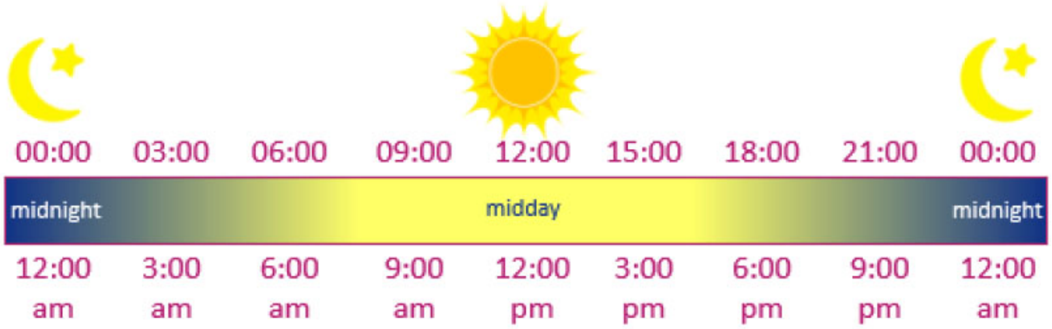

Example: 8:30 AM

16. 2) During the past month, how long (in minutes) has it usually taken you to fall asleep each night? *

NUMBER OF MINUTES

17. 3) During the past month, what time have you usually gotten up in the morning? *

GETTING UP TIME

Example: 8:30 AM

18. 4) During the past month, how many hours of actual sleep did you get at night? *
(This may be different than the number of hours you spent in bed.)

HOURS OF SLEEP PER NIGHT

Mark only one oval.

- 1
- 2
- 3
- 4
- 5
- 6
- 7
- 8
- 9
- 10
- 11
- 12
- 13
- 14
- 15
- 16
- 17
- 18
- 19
- 20
- 21
- 22
- 23
- 24

19. 5) During the past month, how often have you had trouble sleeping because you . *

Mark only one oval per row.

	Not during the past month	Less than once a week	Once or twice a week	Three or more times a week
a) Cannot get to sleep within 30 minutes	⬭	⬭	⬭	⬭
b) Wake up in the middle of the night or early morning	⬭	⬭	⬭	⬭
c) Have to get up to use the bathroom	⬭	⬭	⬭	⬭
d) Cannot breathe comfortably	⬭	⬭	⬭	⬭
e) Cough or snore loudly	⬭	⬭	⬭	⬭
cold	⬭	⬭	⬭	⬭
hot	⬭	⬭	⬭	⬭
h) Had bad	⬭	⬭	⬭	⬭
	⬭	⬭	⬭	⬭

20. Other reason(s), please describe and How often during the past month have you had trouble sleeping because of this

____

____

____

____

____

21. 6) During the past month, how would you rate your sleep quality overall? *

Mark only one oval.

- Very good
- Fairly good
- Fairly bad
- Very bad

22. *

Mark only one oval per row.

	Not during the past month	Less than once a week	Once or twice a week	Three or more times a week
7) During the past month, how often have you taken medicine to help you sleep (prescribed or "over the counter")?				
8) During the past month, how often have you had trouble staying awake while driving, eating meals, or engaging in social activity?				

23. 9) During the past month, how much of a problem has it been for you to keep up enough enthusiasm to get things done? *

Mark only one oval.

- No problem at all
- Only a very slight problem
- Somewhat of a problem
- A very big problem

24. 10) Do you have a bed partner or room mate?

Answer is not mandatory

Mark only one oval.

- No bed partner or room mate
- Partner/room mate in other room
- Partner in same room, but not same bed
- Partner in same bed

25. 11) If you have a room mate or bed partner, ask him/her how often in the past month you have had .

Answer is not mandatory

Mark only one oval per row.

	Not during the past month	Less than once a week	Once or twice a week	Three or more times a week
Loud snoring	◯	◯	◯	◯
Long pauses between breaths while asleep	◯	◯	◯	◯
Legs twitching or jerking while you sleep	◯	◯	◯	◯
Episodes of disorientation or confusion during sleep	◯	◯	◯	◯
Other restlessness while you sleep	◯	◯	◯	◯

Internet Gaming Disorder Scale

26. *

Mark only one oval per row.

	Yes	No
**1) Do you feel preoccupied with your gaming behavior?**	⬭	⬭
**2) Do you feel more irritability, anxiety or even sadness when you try to either reduce or stop your gaming activity**	⬭	⬭
**3) Do you feel the need to spend increasing amount of time engaged gaming in order to achieve satisfaction or pleasure**	⬭	⬭
**4) . Do you systematically fail when trying to control or cease your gaming activity?**	⬭	⬭
**5) Have you lost interests in previous hobbies and**	⬭	⬭

other entertainment activities as a result of your engagement with the game		
6) Have you continued your gaming activity despite knowing it was causing problems between you and other people?		
7) Have you deceived any of your family members, therapists or others because the amount of your gaming activity		
8) Do you play in order to temporarily escape or relieve a negative mood (e.g., helplessness, guilt, anxiety)?		
9) Have you jeopardized or lost an important relationship, job or an educational or career opportunity		

opportunity because of your gaming activity

This content is neither created nor endorsed by Google.

Google Forms

DISCLAIMER: The above article has been published, as is, ahead-of-print, to provide early visibility but is not the final version. Major publication processes like copyediting, proofing, typesetting and further review are still to be done and may lead to changes in the final published version, if it is eventually published. All legal disclaimers that apply to the final published article also apply to this ahead-of-print version.
